# Toward the utilisation of resources in space: knowledge gaps, open questions, and priorities

**DOI:** 10.1038/s41526-023-00274-3

**Published:** 2023-03-25

**Authors:** Jan Cilliers, Kathryn Hadler, Joshua Rasera

**Affiliations:** 1 Department of Earth Science and Engineering, Imperial College London, Exhibition Road, London, SW7 2AZ United Kingdom; 2 European Space Resources Innovation Centre (ESRIC), Luxembourg Institute of Science and Technology (LIST), Maison de l’Innovation, 5, avenue des Hauts-Fourneuax, Esch-sur-Alzette, L-4362 Luxembourg

**Keywords:** Engineering, Scientific community

## Abstract

There are many open science questions in space resource utilisation due to the novelty and relative immaturity of the field. While many potential technologies have been proposed to produce usable resources in space, high confidence, large-scale design is limited by gaps in the knowledge of the local environmental conditions, geology, mineralogy, and regolith characteristics, as well as specific science questions intrinsic to each process. Further, the engineering constraints (e.g. energy, throughput, efficiency etc.) must be incorporated into the design. This work aims to summarise briefly recent activities in the field of space resource utilisation, as well as to identify key knowledge gaps, and to present open science questions. Finally, future exploration priorities to enable the use of space resources are highlighted.

## Introduction

The use of space resources is critical for the future of long-term and deep-space exploration. Space exploration presents challenges for sustainability; single-use launchers, non-refuelable satellites, and a need for all hardware and consumables to be supplied from Earth, all add appreciable resource use and cost to space programmes. Fortunately, significant progress is being made: SpaceX are Blue Origin are demonstrating the value of re-usable launch systems^1^; on-orbit refuelling is being developed by start-ups such as Orbit Fab and Orbital Express, as well as established actors, such as Airbus and Busek^2^.

The use of space resources to provide propellant, habitation and materials critical to support human life (e.g. water, oxygen) will unlock the full potential of space exploration, enabling humans to travel further and spend longer in space^3–5^. This will transform the economics of space exploration.

The use of space resources, known as in situ resource utilisation (ISRU), or more generally as space resource utilisation (SRU), is not a new concept. A detailed history of SRU is provided by Meurisse and Carpenter^6^. In brief, the utilisation of space resources was first suggested by Konstantin Tsiolkovsky, widely considered the originator of modern approaches to rocketry, in 1903^7,8^. Lunar SRU was proposed by Clarke^9^ in the 1950s. During the Apollo Era in the 1960s, SRU was suggested by Carr^10^ as a practical means to reduce launch mass and terrestrial dependency. In the subsequent 50 years, the concept has grown in maturity. Many terrestrial studies have been undertaken to design and test candidate technologies (e.g., refs. ^11–17^).

As of 2022, SRU has been demonstrated only once in space, despite these technologies playing an key role in ESA’s and NASA’s space exploration road maps^12,18^. The MOXIE (*M*ars *OX*ygen *I*SRU *E*xperiment) payload on board NASA’s Perseverance Rover produced oxygen from Mars’ CO_2_-rich atmosphere in 2021 by solid oxide electrolysis^19^. Lunar SRU demonstration missions are under development (e.g., refs. ^20,21^), and preliminary missions to test new SRU legal and economic frameworks are scheduled throughout 2023, for example ispace inc.’s HAKUTO-R Mission 1, currently en route to the Moon^22,23^.

Today, accessing and using space resources is a focus of many space agencies^18,24–27^, governments^28–31^, intergovernmental organisations^32,33^, and private industry^34–36^. More recently, there has been renewed interest in SRU for a number of applications, such as:

- Producing oxygen and metals on the Moon and Mars (e.g. refs. ^19,37–46^);
- Extracting water from the lunar poles (e.g. refs. ^47–51^);
- Extracting water, volatiles and metals from near-Earth objects (e.g. refs. ^52–58^);
- Construction of habitats and thermal shelters, including by additive manufacturing (e.g. refs. ^59–70^); and,
- The manufacture of equipment and technology from local resources (e.g. refs. ^71–76^).

Demonstration-scale SRU projects are a viable, necessary first step for the industry. Their success will broaden appreciably the knowledge base of the SRU and lunar science communities. Detailed knowledge of the local geology, mineralogy and regolith characteristics will enhance greatly confidence in the designs of mining, extraction and production systems at an industrial scale. Other science questions, intrinsic to each specific process, should be addressed to optimise the design of industrial-scale systems. Both the environmental operating conditions (e.g., local electrostatic and radiation environments) and engineering constraints (e.g. energy use, required throughput, expected efficiency, etc.) will affect equipment designs significantly^77^). The success of large-scale resource utilisation processes is dependent therefore on a sufficient knowledge of the specific resource and region of interest, as well as the technology capability required to extract useful products.

This work was developed following the European Space Agency’s SciSpacE Space Resources White Paper exercise. Here, knowledge gaps, open science questions, and research priorities for the lunar science and SRU communities are identified. As the capabilities and limitations of SRU are clarified through in situ demonstrations, it will be possible to address many of these gaps and questions, and in doing so, will improve greatly the development of large-scale SRU technologies. Furthermore, answering these questions will provide tremendous value to the scientific community.

## The SRU process

The extraction and use of space resources is analogous to the extraction and use of terrestrial resources^78,79^. First, the given resource (e.g. oxygen, water ice) must be identified through prospecting and ground truth exploration to increase certainty^80,81^. The composition of the surrounding material and the characteristics of the specific resource within that host material must be understood. The variability in the distribution of the resource in a given region is also required. For example, water ice present within regolith or buried under regolith at the lunar poles varies both spatially and by depth^50,82^. Adopting suitably modified terrestrial industry standards and best practices for exploration and reporting (e.g., JORC and LORS^81^), as well as common terminology^78^ will encourage participation of, and attract investment from non-space actors in SRU.

The chain of technologies linked together to process a particular ore body on Earth is described by a flowsheet^78^. The flowsheet can be subdivided broadly into three key stages: excavation, beneficiation, and extraction of the final product^78^. Excavation has been explored thoroughly in the literature^83^, as have extraction methods^84^. Beneficiation is the process in which mined material is broken or agglomerated and classified by size into a range suitable for further processing, and also to concentrate one component of interest (e.g. water or ilmenite) by physical removal of undesired components. The beneficiation of mined space material into a form suitable for extraction of the require final product has been studied far less in comparison^85^.

In terrestrial mining, the resource, the surrounding material, the location, and the technology used to extract the resource are matched in the process flowsheet such that either:

- The specific resource and its location are targeted depending on available technology; or,
- The technology is designed to meet the extraction requirements of a specific target resource.

Demonstration missions to prove SRU technologies and to raise TRLs have immense value for characterising the potential inputs to the flowsheet. However, the characteristics of resource host material on the Moon, Mars or elsewhere in space are also key inputs to flowsheet design. The processing technologies required must be chosen to maximise confidence in the production levels of the resource as well as the overall operational efficiency. It is inappropriate to assume that a ‘one size fits all’ approach to excavation, beneficiation and extraction would be suitable for SRU. Terrestrial mining operations select carefully the mining equipment used based on the characteristics of the target resource; a SRU will benefit undoubtedly from adopting a similar approach.

Space resource utilisation requires engineering solutions to produce a reliable supply of usable products from a naturally variable feedstock^77^. The use of mineral resources for SRU remains untested anywhere in space, however this will change in the coming years with demonstration missions (e.g. PROSPECT), the exploration of the lunar poles, and NASA’s upcoming regolith collection missions^20,22^. For SRU to become a realisable option for future space travel, it will be important for early demonstration missions to address as many open science questions as possible, as this will enable ultimately the implementation of SRU at an industrial scale.

## Data: the key knowledge gap

There remain many aspects of SRU that are poorly quantified, through lack of available data and samples, and limitations with demonstrating space technologies on the surface of the Earth. The data required to enable SRU in the future can be categorised into two groups: environmental data and resource data. Such data will further have intrinsic scientific value.

Environmental data are critical for the development of robust equipment with high operational availability and long-term usage in mind. Deep knowledge of the local environmental conditions will impact directly the design choices made to ensure that only the most robust and reliable technologies are deployed. The operating environment will affect significantly the design and operation of any process, for example:

- Variation in the electrostatic properties of regolith under different conditions (e.g. day and night);
- Designing operations for lower gravity, different atmospheric characteristics, or no atmosphere at all;
- Designing to withstand extremely high and low temperatures, and the process of cycling through them;
- Material handling in dusty environments;
- Local radiation environment; and,
- Designing for reliability and durability.

Resource data are imperative for selecting appropriate technologies for SRU operations. These data must specify:

- The location of the resource;
- The resource properties (e.g. concentration, phase, associations);
- The host material properties (e.g. regolith mineralogy, particle size distribution, particle shape, geotechnical properties);
- The variability in the resource and host material properties (by region, by location and by environmental conditions); and,
- The effect of the resource properties on utilisation (e.g. reactor efficiency, construction strength).

To bridge these gaps, high resolution orbital data sets must be captured and correlated to ground-truth exploration activities at select targets. As an illustration, of the proposals that have been developed previously for large-scale exploitation of resources, several have focused on the extraction of water ice at the lunar poles for propellant production (e.g., refs. ^17,47,48^). These detailed elaborations of production facilities on the Moon are based on assumptions about the form, quantity, variability, and behaviour of icy regolith. At present, there is no ground truth data to verify any of these assumptions, and there are major uncertainties associated with them^86^. Rigorous prospecting and ground truth exploration must be performed in order to raise the level of geological certainty^80,81^. This is standard practice on Earth for the economic development of mines, and will be equally relevant for SRU^80,81^.

The regolith samples returned by the Apollo and Luna missions of the 1960s and 1970s have incredible value for testing bench scale apparatuses, however the amount of lunar material made available for testing is insufficient to develop industrial-scale equipment. Furthermore, the successful development of terrestrial SRU demonstrators will be dependent on the availability of suitable simulants. However, the scientific community, along with private and public sector actors, must agree on a standardised approach for the characterisation of lunar regolith and lunar regolith simulants. Such a standard would enable honest, transparent, like-for-like comparisons of feedstocks and equipment performance, as well as provide justification for using certain simulants for any given technology demonstration.

## Open science questions

There are many open science questions in space resource utilisation due to the novelty and relative immaturity. The following open questions are focused specifically on the applied science aspects needed upscale SRU to an economically viable, industrial scale. One of the benefits of this field is that, with careful design, data and samples required to design SRU processes can be used also to answer open questions of interest to the lunar science community.

- **Which resource characteristics are required to establish the viability of a resource?** This encompasses characteristics of the specific resource such as concentration and occurrence, in addition to those of the host material. Regolith properties, such as size distribution, texture, cohesiveness, electrostatic charge and mineralogy, will be of interest^85,86^. The minimum amount of data to increase the geological certainty of a deposit and how it is collected should also be considered^77,78,80,81^. The use of such datasets in fundamental scientific studies (e.g. geology, planetary evolution) should be a key factor in extra-terrestrial mine planning.
- **How have geological and environmental processes affected properties of resources and how do these properties affect extraction processes?** Environmental factors include geological processes (e.g. volcanism, crustal formation), impacts (delivery of resources *versus* loss of resources during impact reprocessing), solar wind and cosmic ray exposure, and magnetic anomalies. There are many fundamental science questions that can be addressed by understanding the geological and environmental processes occurring in the region of a given space resource, for example impact rate to create local regolith environment. For space resource applications, however, these processes will affect the composition and characteristics of the resource and the host material (e.g. burial depth, porosity, agglutinate content)^87–89^. Geotechnical properties, for example, are affected by the geological makeup (mineralogy, chemistry), impact and space exposure history of the lunar regolith^90^.
- **How do the local environmental conditions affect the resource and potential operations?** For example, electrostatic charging of regolith, gravity, thermal conditions, atmospheric conditions, and radiation. Electrostatic charging of lunar regolith is known to present operational challenges, particularly with regards to reliability^91–94^. It is not possible to replicate simultaneously all aspects of the lunar environment on Earth, and while rapid developments are being made in the field of regolith simulants^95–97^, the production of agglutinates remains difficult at any scale^98^. Questions remain on the magnitude and distribution of electrostatic charging of regolith, and on how this can be mitigated. In situ studies are critical to enhance understanding. Another aspect of interest is the rate of change of environmental conditions (e.g. the atmosphere of Mars).
- **What is the variability of resources in a target region and the effect on processing and extracted product variability?** Variability is an aspect of resource use that is critical in the long term. Variability in the resource and the host material affects every step of the process, from excavation through to purification of the final product^77,99^. Additionally, an understanding of the geological processes, as highlighted previously, will enable better prediction of the resource variability.
- **What are the physical and chemical processes that can be applied to extract and process local resources?** Many processes have been proposed^83–85^, however not all are appropriate for all locations (e.g., hydrogen reduction in the lunar highlands^100^). Strategies for establishing either the most suitable location or the most suitable process are required. Consideration also must be given to the effect of local conditions on process efficiency; this includes feedstock characteristics. End-to-end processing of the resource, including waste disposal/re-use and product storage are also required.

## Outlook and summary

The confident design and successful operation of large- or industrial-scale SRU process operations requires detailed knowledge of the specific resource of interest and suitable extraction technologies. The priority for near-term demonstration missions and future exploration programmes must be to gather high-resolution, high-fidelity data about the performance characteristics of equipment, the local environmental conditions, and the availability of target resources. The terrestrial mining sector has immense expertise in resource exploration; combining this knowledge base with that of lunar/planetary scientists will enable the development of a realistic strategy, fulfilling both scientific goals and enabling SRU. Further, an extensive core and ancillary technology development programme, including optimisation and performance evaluation, is required. This will, in turn, improve the design and development of robust SRU technologies whilst contributing invaluable knowledge to the scientific community.
